# Characterization of VRC01, a potent and broadly neutralizing anti‐HIV mAb, produced in transiently and stably transformed tobacco

**DOI:** 10.1111/pbi.12137

**Published:** 2013-11-21

**Authors:** Audrey Y‐H. Teh, Daniel Maresch, Katja Klein, Julian K‐C. Ma

**Affiliations:** ^1^ Molecular Immunology UnitInfection and Immunity Research CentreSt. George's University of LondonLondonUK; ^2^ Department of ChemistryUniversity of Natural Resources and Applied Life SciencesViennaAustria; ^3^ Department of Infectious DiseasesDivision of MedicineImperial College LondonLondonUK

**Keywords:** VRC01, HIV‐1 broadly neutralizing antibody, transient expression, stable transgenics, pTRAk.2

## Abstract

The proposed clinical trial in Africa of VRC01, a potent broadly neutralizing antibody (bNAb) capable of neutralizing 91% of known HIV‐1 isolates, raises concerns about testing a treatment which will be too expensive to be accessible by the most important target population, the poor in under‐developed regions such as sub‐Saharan Africa. Here, we report the expression of VRC01 in plants as an economic alternative to conventional mammalian‐cell‐based production platforms. The heavy and light chain genes of VRC01 were cloned onto a single vector, pTRAk.2, which was transformed into *Nicotiana benthamiana* or *Nicotiana tabacum* using transient and stable expression production systems respectively. VRC01 has been successfully expressed transiently in plants with expression level of approximately 80 mg antibody/kg; stable transgenic lines expressing up to 100 mg antibody/kg were also obtained. Plant‐produced VRC01 from both systems showed a largely homogeneous *N*‐glycosylation profile with a single dominant glycoform. The binding kinetics to gp120 IIIB (approximately 1 nm), neutralization of HIV‐1 BaL or a panel of 10 VRC01‐sensitive HIV‐1 Env pseudoviruses of VRC01 produced in transient and stable plants were also consistent with VRC01 from HEK cells.

## Introduction

Human immunodeficiency virus infection/acquired immunodeficiency syndrome (HIV/AIDS) is a global pandemic and one of the leading causes of deaths in the world (WHO, 2008). The worst affected region is sub‐Saharan Africa, accounting for nearly 70 per cent of all people living with HIV. There is currently no cure or effective vaccine for HIV/AIDS. Current treatment protocols involve antiretroviral therapy (ART), a combination of three or more antiretroviral (ARV) drugs which control viraemia, thus increasing the survival rate of HIV‐infected individuals (May and Ingle, 2011). ARV drugs can also be used to reduce the chance of HIV transmission through ‘treatment as prevention’ (TasP) (WHO, 2012a). Otherwise, ART can be used by noninfected individuals topically as vaginal or rectal microbicides (Shattock and Rosenberg, 2012) or as pre‐ or postexposure prophylaxis (PrEP or PEP) (WHO, 2012b; WHO and ILO, 2007).

Life‐long treatment or prevention using ARV drugs is expensive, between US$10 000 and US$12 000 per year per person in the USA (Bartlett, 2006) as it involves sustained periods of drug delivery. Worldwide treatment coverage of people requiring access to ART, currently at 54 per cent (UNAIDS, 2012), still falls below the target goal of 80 per cent (UN, 2011; WHO, UNAIDS and UNICEF, 2007). Moreover, long‐term use of ARV drugs can also result in toxicities with unpredictable effects on health as well as development of drug resistance and viral rebound if the treatment regimen is not adhered to (Barbaro, 2006; Barbaro *et al*., 2005; Chen *et al*., 2013; Rusconi *et al*., 2007). Therefore, the search continues for additional therapeutic modalities to complement or replace current ARV drugs (Arts and Hazuda, 2012).

Broadly neutralizing antibodies (bNAb) constitute a rare class of antibodies which are effective against a broad range of HIV strains (Doria‐Rose *et al*., 2010; Li *et al*., 2007, 2009; Simek *et al*., 2009). They naturally develop in 15%–25% of individuals after many years of infection. Most of the neutralizing antibodies, including bNAbs, disrupt viral replication by immune exclusion but there are also evidence to suggest viral neutralization by Fc‐mediated antibody activities (Overbaugh and Morris, 2012). Since 2009, a new generation of bNAbs, which are more potent and broadly neutralizing compared with previous HIV‐1 mAbs, has been identified (see Chen *et al*., 2013 for review). This new generation of bNAbs holds much promise for HIV‐1 therapy and prevention. Recently, Klein *et al*. (2012) has shown that a combination of five new‐generation bNAbs can effectively control HIV‐1 infection and suppress viral load to levels below detection in a humanized mice model. The antibody cocktail has longer half‐life compared with current ARV drugs and can control viraemia for an average of 60 days after therapy was discontinued. There are also plans for a clinical trial to test the new‐generation bNAbs to prevent the spread of HIV‐1 in high‐risk adult males (Wadman, 2013).

VRC01, one of these new generation broadly neutralizing IgG1, was obtained through the use of resurfaced antigenic probes to select for B cells (obtained from a HIV‐1 infected donor) that express bNAbs directed at HIV‐1 CD4 binding sites (Wu *et al*., 2010). It blocks viral entry by partially mimicking the interaction of the CD4 receptor with HIV‐1 gp120 envelope glycoprotein (Zhou *et al*., 2010). *In vitro* neutralization tests have shown that VRC01 is capable of neutralizing 91% of known HIV‐1 isolates (Wu *et al*., 2010). The antibody, when applied as a topical gel, also protected against HIV‐1 infection in vaginally challenged humanized mice (Veselinovic *et al*., 2012). Moreover, in the preclinical testing conducted in nonhuman primates, preliminary results showed that animals receiving VRC01 were protected against mucosal challenge with a chimeric simian‐human immunodeficiency virus (SHIV) compared with the control group (Pegu *et al*., 2011).

An investigational new drug application to the US Food and Drug Administration is currently underway for VRC01 (Lopez *et al*., 2013). The investigators proposed that VRC01 will be first safety‐tested in adults and infants in the USA. It was then hoped that subsequent trials will be carried out in African countries such as Malawi, Tanzania and Uganda to investigate VRC01 as a prophylactic to complement ART in the prevention of mother‐to‐child transmission of HIV‐1 via breastfeeding. The proposal for the clinical trial raised issues about testing a treatment which would not be affordable to its target population in low‐income countries, as monoclonal antibody therapies are usually very expensive (Lopez *et al*., 2013; Wadman, 2013).

Plants offer an attractive alternative to conventional mammalian‐cell‐culture‐based platforms for recombinant protein production, including monoclonal antibodies. In recent years, technology developments have resolved early difficulties with expression levels (Fischer *et al*., 2012). Furthermore, industry and regulatory concerns regarding cGMP compliant manufacture for human use have been addressed, with regulatory approvals for clinical trials in the UK (J. Ma, pers. comm.) and the USA (J. Butler, pers. comm.). Here, we compare transient and stable transgenic plant production platforms to manufacture new‐generation bNAbs against HIV‐1, using VRC01 as an example. VRC01 light and heavy chain genes were cloned into a single vector and transformed into tobacco plants. We report the successful production of VRC01 in tobacco using both methods. The plant‐produced VRC01 antibodies possess a largely homogenous glycosylation pattern, and we have also investigated their binding kinetics. Furthermore, VRC01 from transiently and stably transformed plants neutralizes HIV‐1 isolates across four different clades with neutralizing breadth and potency consistent with VRC01 from HEK cells. These developments now offer hope that the specific advantages of plant production platforms—namely low manufacturing entry cost, large scalability and simple technology amendable to transfer to developing countries—may soon be realized.

## Results

### Determination of antibody accumulation levels

Plant‐codon‐optimized VRC01 light and heavy chain genes (GeneArt) were cloned into the binary expression vector pTRAk.2 (van Dollerweerd, C.J., Teh, A.Y‐H., Banyard, A.C., Both, L., Lotter‐Stark, H.C.T., Tsekoa, T., Phahladira, B., Shumba, W., Sabeta, C.T., Szeto, T.H., Gruber, C., Fooks, A.R., Chikwamba, R.K. and Ma, J.K‐C., in preparation).

For transient expression, *Agrobacterium* harbouring the T‐DNA constructs were delivered into *Nicotiana benthamiana* via vacuum infiltration (Kapila *et al*., 1997). Twelve individual plants were used. The expression levels of 6‐day postinfiltrated (dpi) *N. benthamiana* leaves (approx. 5–7 g) from each plant were determined using an ELISA designed to detect fully assembled IgG. The experiment was performed twice. The average expression level was 81 mg/kg fresh weight VRC01 mAb before purification.

pTRAk.2‐VRC01 was also used to generate stably transformed plants through *Agrobacterium* co‐cultivation of *Nicotiana tabacum* cv. Petit Havana SR‐1 leaf discs (Horsch *et al*., 1985). Thirty‐five primary transformants (T_0_) were tested using ELISA set up to detect only full‐length IgGs. The highest producing expressed at ~100 mg/kg fresh weight (data not shown). This T_0_ plant line was self‐fertilized to generate T_1_ plants. 30 T_1_ plants were screened and the expression levels averaged about 130 mg/kg (unpurified; Table 1). The yield (before purification) of the VRC01 obtained from the stably transformed T_1_ generation was not significantly different (*P *<* *0.05) from the expression levels from *N. benthamiana* plants transiently transformed with pTRAk.2 (Table 1).

**Table 1:** Expression levels (before purification) of VRC01 in transient (*n* = 24) and stable (*n* = 30) expression systems. Results are expressed as mean ± SD (in mg/kg fresh weight)

Vector	Host	Production system	Yields (mg/kg)
pTRAk.2	*N. benthamiana*	Transient	81 ± 17 (*n* = 24)
pTRAk.2	*N. tabacum* cv. Petit Havana SR‐1	Stable	129 ± 76 (*n* = 30^a^)

a T1 generation from self‐crossed primary transformant.

### PAGE and Western blot analysis of mAb integrity

**Figure 1:**
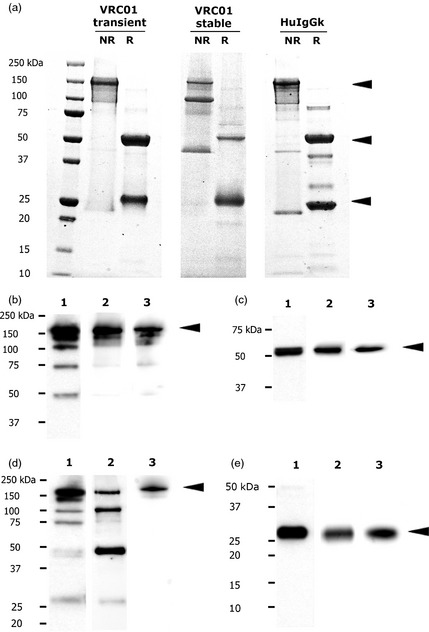
Expression profiles of VRC01 produced in plants and HEK cells. (a) Coomassie‐stained SDS‐PAGE gel showing VRC01 purified from transient plants (left panel) and stable transformants (T_0_ generation; middle panel) under nonreducing (NR) and reducing (R) conditions. Commercially available human IgG1 kappa from human serum (Sigma) was used as positive control (right panel). Five micrograms of purified protein was loaded onto each lane. Full‐length IgGs on nonreducing gels, as well as the heavy and light chain on reducing gels, are indicated by black arrows. (b–e) Nonreducing (b and d) and reducing (c and e) Western blot analysis of VRC01 in 1) transient plant crude extract, 2) crude extract from stable transformants (T_0_ generation) and 3) VRC01 purified from HEK cells. The heavy chain was detected using a polyclonal goat anti‐human IgG (Fc fragment) antiserum (b and c), and the light chain was detected using a goat anti‐human IgG kappa chain antibody (d and e). Full‐length IgG, as well as heavy and light chains, are indicated by black arrows.

Coomassie‐stained SDS‐PAGE gels of VRC01 purified from transient and transgenic plants are shown in Figure 1a. In both cases, there is a major band at ~150 kDa in the nonreduced samples that corresponds to the full‐length IgG band identified in the positive control human IgG sample (top arrow). The identity of this band was confirmed by Western blot, as it was detected by both antiheavy and light chain antisera (Figure 1b, d). In all these antibody samples (transient, stable and positive IgG control), there were numerous smaller bands, which are likely to be degradation products as they were also immunoreactive in the Western blot. The stably expressed VRC01 appeared to demonstrate slightly more degradation, with prominent bands at ~100 kDa and ~50 kDa. Both of these bands were reactive with the antilight chain reagent in Western blot (Figure 1d). Under reducing conditions, the bands resolved into a ~50‐kDa band and a ~25‐kDa band (Figure 1a). Identical bands were observed for the HEK‐cells‐derived version of VRC01. These bands were identified as heavy and light chains by Western blotting (Figure 1c, e).

### Glycopeptide analysis

**Figure 2:**
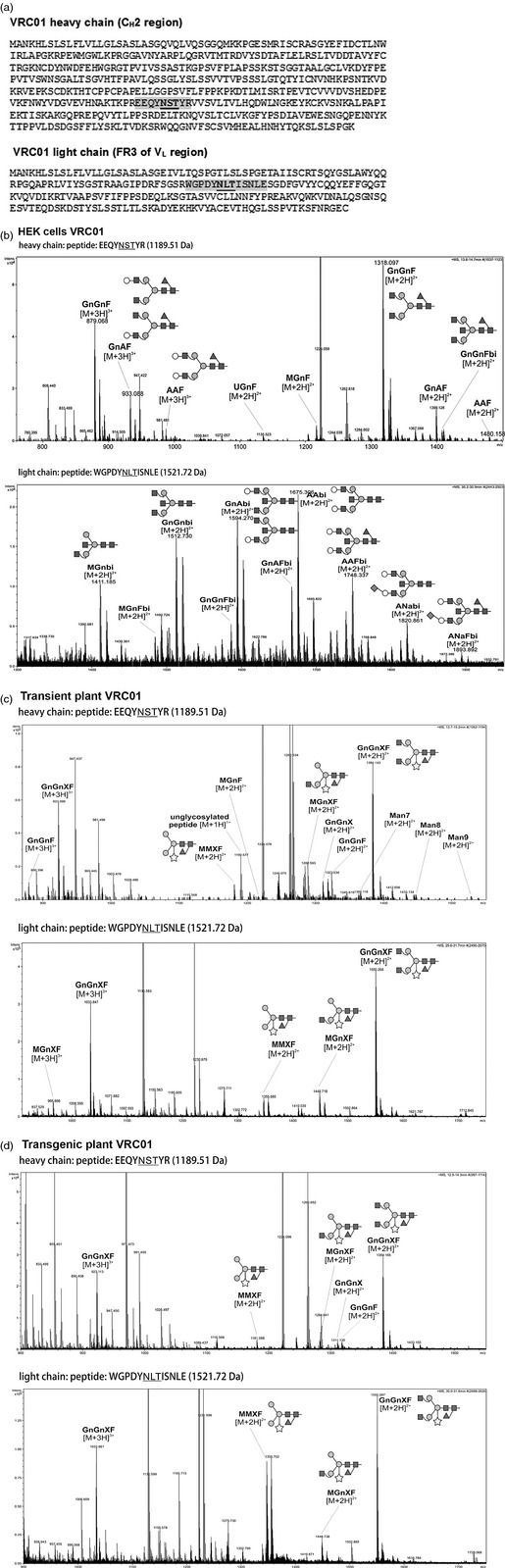
Characterization of *N*‐glycan population of VRC01 from HEK cells, transient plants and transgenic plants. (a) Amino acid sequence of VRC01 heavy (above) and light chain (below). The highlighted portion represents the glycopeptide fragment generated after trypsin/GluC digest and subsequently analysed using LC‐ESI‐MS. Potential *N‐g*lycosylation sites are underlined: the heavy chain has an Asn/Ser/Thr consensus sequence in the C_H_2 region; the light chain has an Asn/Leu/Thr consensus sequence in the FR3 of V_L_ region. (b, c and d) Deconvoluted glycopeptide spectra of VRC01 derived from (b) HEK cells, (c) transiently transformed *Nicotiana benthamiana* plants and (d) transgenic *Nicotiana tabacum* plants as determined by LC‐ESI‐MS after *S*‐carbamidomethylation and trypsin/GluC digest. The glycopeptides analysed were EEQYNSTYR (1189.51 Da) in the heavy chain (top panel) and WGPDYNLTISNLE (1521.72 Da) in the light chain (bottom panel; glycosylation sites are underlined). Marked peaks correspond to glycans with different *m/z* values. Diamonds, white circles, dark squares, grey circles, triangles and stars represent *N‐*acetylneuraminic acid, galactose, *N*‐acetylglucosamine, mannose, fucose and xylose, respectively.

The analysis of heavy and light chain sequences of VRC01 using EssembleGly (Caragea *et al*., 2007) revealed two potential *N*‐linked glycosylation sites: one in the heavy chain C_H_2 region at N_297_ and one in the FR3 of the light chain V_L_ region (Figure 2a).

Glycan addition at both sites in VRC01 derived from HEK cells, transient plants and transgenic plants were confirmed using liquid chromatography‐electrospray ionization‐mass spectrometry (LC‐ESI‐MS) after *S‐*carbamidomethylation and trypsin/GluC digest (Figure 2b–d). The deconvoluted spectra of the glycopeptide elution range were used for identification of *N*‐glycan structures. HEK‐cells‐derived VRC01 was shown to have GnGnF^6^ as the predominant glycan structure on the heavy chain, with lesser amounts of GnAF^6^, AAF^6^ and GnGnF^6^bi structures (Figure 2b, upper panel, nomenclature as used at www.proglycan.com). On the light chain, there was greater diversity of glycans with at least ten structures identified (Figure 2b, lower panel).

In contrast, the plant versions of VRC01 (which were similar), both have GnGnXF^3^ as the major glycan structure, with MGnXF^3^ and MMXF^3^ as the two other major glycans (Figure 2c, d). This was observed in both the heavy and light chains. For the light chain, the MMXF^3^ glycan was slightly more prevalent in stable expression of the antibody (Figure 2d, lower panel).

### HIV‐gp120 binding kinetics

**Figure 3:**
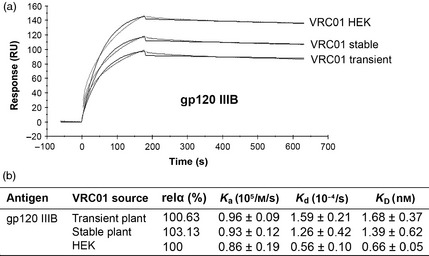
Binding kinetics of VRC01 from plant and HEK cells to HIV gp120 IIIB. (a) SPR sensorgram comparing the interaction of Protein‐A captured VRC01 from transient plants (VRC01 transient), stably transformed plants (VRC01 stable) and HEK cells (VRC01 HEK) with recombinant gp120 III. Protein A was immobilized on a CM5 chip, and VRC01 antibodies were captured to ≈100RU. gp120 were injected at a concentration of 20 μg/mL. Experimental curves are shown as grey lines and fitted curves shown in black. Sensorgram shown represents one of three similar experiments. (b) Binding kinetics: association constant (*K*_a_), dissociation constant (*K*_d_) and affinity (*K*_D_) are shown for VRC01 from three sources. Each assay was performed in triplicate. Error bars denotes ± SD.

To evaluate the antigen‐binding kinetics of plant‐produced VRC01, binding of HIV‐1 envelope glycoprotein gp120 IIIB to VRC01 mAb captured on a protein A‐coated CM5 sensor chip was measured by surface plasmon resonance (SPR). A preliminary direct ELISA confirmed that plant‐derived VRC01 mAb binds to gp120 (data not shown). Protein A was first immobilized onto a CM5 sensor ship by amine coupling. Then, plant‐ or HEK‐cells‐derived VRC01 was captured on to the chip surface. Purified recombinant gp120 was injected over the chip at 20 μg/mL. The binding response of VRC01 from transient and stable plants as well as from HEK cells to gp120 molecules was fitted with a 1 : 1 Langmuir model after subtraction of the response from the blank flow cell and protein A‐antibody only controls (Figure 3a). The equilibrium dissociation constant (*K*_D_) of the interaction between plant VRC01 and gp120 was calculated to be 1.68 ± 0.37 and 1.39 ± 0.62  nm, respectively, for transient and stable plant‐derived VRC01 compared with 0.66 ± 0.05 nm from VRC01 from HEK cells (Figure 3b). The association and dissociation constants of the SPR analyses are also shown in Figure 3b. There were no significant differences between the results (*P *<* *0.05). Furthermore, there were no differences in relative antigen‐binding activity (relα) between the two plant‐derived VRC01 and HEK‐cells‐derived VRC01.

### TZM‐bl‐neutralizing assays

**Figure 4:**
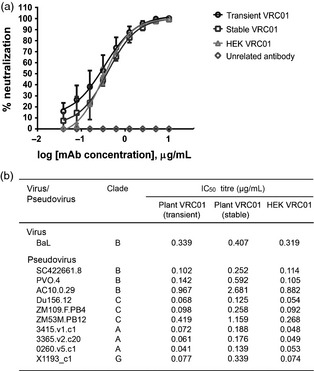
Neutralization of HIV‐1 virus and pseudoviruses by VRC01 produced in plants and HEK cells. (a) Neutralization curves of transient plant‐ (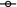), stable transgenic plant ‐(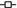) and HEK cells ‐(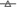)produced VRC01 against HIV‐1BaL; tested using TZM‐bl cells. A nonspecific plant‐derived antibody was used as negative control (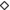). Error bars denotes ± SD. Each assay was repeated three times. (b) Neutralization IC_50_ values (μg/mL) of VRC01 produced in transient and stable transgenic plants as well as HEK cells against HIV‐1 BaL (clade B) and 10 VRC01‐sensitive HIV‐1 Env pseudoviruses from clade A, B, C and G; tested using TZM‐bl cells.

We initially performed HIV neutralization assays comparing VRC01 produced in transient and stable plants and in HEK cells in inhibiting TZM‐bl cell infection by HIV‐1 BaL. The antibodies were first incubated with HIV‐1 BaL at a range of concentrations before being added to TZM‐bl cells containing the HIV Tat‐regulated luciferase reporter gene. Virus neutralization by the antibodies was measured as a function of a reduction in the expression of the luciferase reporter gene. VRC01 mAbs from plants and HEK cells showed similar neutralizing activity (Figure 4a), with IC_50_ of 0.339, 0.407 and 0.319 μg/mL for VRC01 from transient plants, stable plants and HEK cells, respectively (Figure 4b). The IC_50_ values of plant‐ and HEK‐cells‐derived VRC01 were not statistically different (*P *<* *0.05).

A more comprehensive analysis using a variety of HIV‐1 clades and isolates was then performed (courtesy of Dr. M. Seaman, through the CAVD). 10 VRC01‐sensitive pseudoviruses from clades A, B, C and G were tested (Figure 4b). The IC_50_ titres of VRC01 from transient plants were concordant with the HEK‐cells‐produced VRC01 mAb. VRC01 from stable plants also neutralized all pseudoviruses, but appeared to be slightly less potent.

## Discussion

Broadly neutralizing antibodies (bNAbs) have different properties compared with small molecule antiretroviral (ARV) drugs and represent an opportunity for alternative or complementary prophylactic and therapeutic approaches. For example, they are highly specific, interact and neutralize the pathogen directly and also possess a longer half‐life *in vivo* (Klein *et al*., 2012). Anti‐HIV bNAbs can be used in conjunction with small molecule ARV drugs in a multidrug combination, similar to antitumour (Scott *et al*., 2012) or antimicrobial (Pachl *et al*., 2006) combined therapy, to counter drug‐resistant HIV‐1 strains and viral escape (Pirrone *et al*., 2011). Furthermore, they can also be used for individuals intolerant to current ARV drugs. However, biologics are costly. The top 15 mAbs and Fc fusion products cost ~$8 000 per gram in 2008 (Kelley, 2009). Prophylactic possibilities include vaginal or rectal microbicides, pre‐exposure prophylaxis (PrEP), postexposure prophylaxis (PEP) and perinatal intervention. Furthermore, if mAbs are to be used in antiretroviral applications, several antibodies might be needed in combination as an antibody cocktail. Considering the long‐term, sustained usage of ARV drugs in the treatment and prevention of HIV/AIDS (WHO, 2006), it is therefore imperative to look for less expensive and more scalable production platforms which can be used to replace or supplement current mammalian‐cell‐culture‐based systems (Beck *et al*., 2010).

Plants were first proposed as a manufacturing platform for monoclonal antibodies over 20 years ago (Hiatt *et al*., 1989). Initial optimism for low‐cost high‐volume production was blunted both by technical limitations of the early technology as well as doubts over public acceptability of the new biotechnology. In addition, acceptance by the industry was affected by widespread concerns over the pharmaceutical regulatory compliance by plant‐based manufacturing systems. The technical issues of recombinant protein expression levels have been addressed through a variety of means (Desai *et al*., 2010), including gene codon optimization (Batard *et al*., 2000; Rouwendal *et al*., 1997), expression vector design (Gleba *et al*., 2007; Sainsbury *et al*., 2009, 2010), improved screening strategies of potential production lines, optimizing plant cultivation (Colgan *et al*., 2010) and plant breeding (Hood *et al*., 2002).

In 2012, the first plant‐derived pharmaceutical product, Elelyso™ by Protalix BioTherapeutics, was approved by the FDA to treat Type I Gaucher's disease (Edney, 2013). Regulatory approvals have also been granted for clinical trials of, among others, recombinant human insulin produced in transgenic safflower, non‐Hodgkins’ lymphoma vaccine (comprising of monoclonal antibodies possessing the B‐cell lymphoma idiotype) expressed transiently in *N. benthamiana*, and in 2011, the anti‐HIV monoclonal antibody 2G12 produced in transgenic *N. tabacum* (Ma, J.K‐C., Drossard, J., Lewis, D., Altmann, F., Boyle, J., Christou, P., Cole, T., Dale, P., van Dolleweerd, C.J., Isitt, V., Katinger, D., Lobedan, M., Mertens, H., Paul, M.J., Rademacher, T., Sack, M., Sparrow, P., Stiegler, G., Stoger, E., Twyman, R.M., Vcelar B. and Fischer R., pers. comm.). It is thus evident that recombinant pharmaceuticals manufacturing in plants are feasible. The focus now shifts back to target products and applications where plant manufacture is most desirable. Although lower cost of goods is a desirable feature of *Nicotiana*‐based manufacturing, the potential for low cost of entry to clinical trials (a result of reduced early capital costs) may be an additional and significant driver. Of equal importance is the potential scalability of manufacture, which opens up prophylactic and/or therapeutic interventions that were previously unattainable.

HIV‐neutralizing antibodies are therefore an obvious target for manufacture in plants. They would almost certainly be required at a very large scale, and cost of goods may become an important consideration for cocktail products containing two, three or more active drug products. Very recently, two groups reported production of VRC01 mAb in plant transient expression systems (Hamorsky *et al*., 2013; Rosenberg *et al*., 2013). Hamorsky *et al*. (2013) devised a novel tobamovirus replicon vector in *N. benthamiana*, while Rosenberg *et al*. (2013) reported transient expression using the same pTRAk vector used here in both *N. benthamiana* and *N. tabacum*. Both groups demonstrated successful expression of functional VRC01 of levels equivalent to those achieved here. In this study, we have demonstrated VRC01 production by stable expression in transgenic plants.

The finding that the expression levels in stable transgenics and transient expression were quite similar was unexpected, as previous literature has generally indicated much higher levels of expression using transient systems (Marillonnet *et al*., 2005; Sainsbury and Lomonossoff, 2008). Interestingly, with a second transient expression system CPMV‐HT (Sainsbury and Lomonossoff, 2008), similar levels of expression were also obtained (data not shown). In the case of HIV bNAbs, the use of a highly efficient transgenic system may be extremely advantageous, because the simplified manufacturing process could be performed in resource‐poor regions, especially those that have an urgent interest in addressing HIV transmission.

The transgenic plant lines generated here expressed VRC01 with no additional sequences (such as the KDEL tags used by Rosenberg *et al*.) or residual amino acids (as left by the kex2p‐like cleavage strategy used by Hamorsky *et al*.). These transgenic plant lines have now entered a breeding programme to achieve homozygosity and establish genetic and phenotypic stability, which is the initial requirement for developing a master seed bank, leading eventually to GMP compliance (EMEA, 2008).

VRC01 has two potential *N‐*glycosylation sites in the heavy chain hinge region and Framework 2 of the light chain. Glycoanalysis of HEK‐cells‐derived VRC01 has not been published and this is the first report of VRC01 glycosylation in plants. We demonstrated that both potential *N‐*glycosylation sites are utilized, in all the expression systems. Expression in plants generally resulted in a less heterogeneous mixture of glycoforms, as compared with HEK cells, with the typical GnGnXF^3^ complex glycan dominant, particularly in transiently expressed VRC01. Mono‐glycosylation of monoclonal antibodies has been demonstrated for one other antibody produced in plants, the anti‐HIV mAb 2G12 (Forthal *et al*., 2010; Sainsbury *et al*., 2010), but not to our knowledge in other expression systems. In terms of product quality control, the ability of plants to produce highly homogeneous drug product could become an important manufacturing advantage. In cell‐mediated anti‐HIV activity, Forthal *et al*. (2010) reported that plant‐produced 2G12 with the GnGnXF^3^ glycoform performed as well as 2G12 produced in CHO‐cells in terms of interaction of the antibody Fc region with FcγRI, FcγRIIa and FcγRIIb receptors as well as antibody‐dependent cell‐mediated virus inhibition (ADCVI). 2G12 with plant‐ or mammalian‐type core fucose did have reduced binding to FcγRIIIa, and its potency in ADCVI assays could be enhanced by removal of the fucose residue. However, Rosenberg *et al*. (2013) showed that the removal of the same core α1,3‐fucose from plant VRC01 did not increase its ADVCI potency. There is no evidence that plant glycans expressed on mammalian antibodies are immunogenic in humans. Plant‐derived 2G12 with the GnGnFX^3^ glycoform was administer vaginally to human volunteers with no adverse effects or immunogenicity (Ma, J.K‐C., Drossard, J., Lewis, D., Altmann, F., Boyle, J., Christou, P., Cole, T., Dale, P., van Dolleweerd, C.J., Isitt, V., Katinger, D., Lobedan, M., Mertens, H., Paul, M.J., Rademacher, T., Sack, M., Sparrow, P., Stiegler, G., Stoger, E., Twyman, R.M., Vcelar B. and Fischer R., pers. comm.). That said, it is encouraging to note the advances that have been made in glycoengineering in plants (Castilho and Steinkellner, 2012; Castilho *et al*., 2013; Strasser *et al*., 2008, 2009) and the potential for producing antibodies with ‘mammalian’ glycans. Interestingly, when mAb 2G12 was expressed with altered glycosylation, there was no significant increase in glycoform heterogeneity (Forthal *et al*., 2010; Strasser *et al*., 2008).

The affinity (*K*_D_) of VRC01 produced in transient and stable plants to the HIV‐1 envelope glycoprotein gp120 IIIB was 1.68 and 1.39 nm, respectively, as determined by SPR analysis. There was no significant difference (*P *<* *0.05) in the binding affinities between plant‐ and HEK‐cells‐derived VRC01. Rosenberg *et al*. (2013) and Hamorsky *et al*. (2013) have also compared the binding of plant‐ and mammalian‐cell‐produced VRC01 to gp120 using capture assays with immobilized mouse anti‐human IgG or protein A. Neither group found any difference in the binding affinities of plant‐ and mammalian‐cell‐produced VRC01. Zhou *et al*. (2010) obtained a *K*_D_ of 5.76 nm when they investigated the binding of HEK‐cells‐produced VRC01 to 93TH057 gp120 using a direct assay. Furthermore, the gp120 binding activities of both plant‐derived VRC01 were very similar to VRC01 from HEK cells. This is in contrast to Hamorsky *et al*. (2013) who reported 75%–83% relative activity of transiently expressed VRC01 when compared to HEK‐cells‐derived VRC01.

Neutralization by plant‐derived VRC01 was compared with HEK‐cells‐derived VRC01 across 11 HIV‐1 virus strains, including representatives from clades A, B, C and G. The results were concordant between VRC01 from transient plant and HEK cells, demonstrating functional equivalence for the plant‐derived antibody. The IC_50_ measured in the pseudotype assays suggest a reduction in potency for VRC01 from stable transformants. This is not likely to reflect qualitative differences between the VRC01 from various sources. The SDS‐PAGE and glycoanalysis suggested increased proteolytic degradation of the stably produced VRC01 sample. Indeed, this sample had been subjected to at least three freeze–thaw cycles before pseudotype neutralization analysis (compared with 1 for the transiently expressed VRC01). No difference in neutralization of HIV‐1 BaL was observed, using material that had undergone two less freeze–thaw cycle. Thus, the slight increase in IC_50_ observed is likely to be due to error in calculation of the true concentration of full‐length, functional IgG in the tested sample. Degradation of antibody might be influenced by the expression platform, but no differences were observed previously when comparing mAb expression by transgenic or transient expression (V. Hehle, PhD thesis, University of London). Edman degradation experiments using three other human monoclonal antibodies from stably transformed plants have suggested that the ~100‐kDa bands are degradation products (Hehle *et al*., 2011; V. Hehle, PhD thesis) likely to represent (Fab’)_2_‐like fragments. The ~50‐kDa band could be light chain dimers. In fact, light chain excess appears to be required for maximal expression of intact full‐length IgG in plants (Nuttall *et al*., 2002). The components of these degradation products may account for the excess of the 25‐kDa band compared with the 50‐kDa band on the reduced PAGE gel (Figure 1a, middle panel). The results have indicated that further work will be needed to finalize a downstream purification process and to characterize the stability in storage of highly purified plant‐derived VRC01. They also highlight the importance of avoiding repeated freeze–thaw cycles.

In summary, the new‐generation bNAb VRC01 was successfully produced using both stable and transient production platforms. This was achieved using a single vector that contains both the light and heavy chain genes under separate promoters. Intact plant‐produced VRC01 was expressed at approximately 80 mg/kg fresh weight using transient expression platforms, and stable lines expressing in excess of 100 mg/kg VRC01 were also obtained. In both cases, the expression levels are significantly higher than that reported for the 2G12 monoclonal antibody that was advanced to phase I clinical trial (www.pharma-planta.net). Further work would be needed to develop appropriate downstream processing/purification protocols, and the efficiency of these protocols will determine the final productivity of the expression system. Plant‐produced VRC01 showed *N*‐glycosylation profiles that were similar to HEK‐cells‐derived VRC01, but more homogeneous. GnGnXF^3^ was the single dominant glycan structure. The binding kinetics of VRC01 produced in stable and transient plants are consistent with values in the published literature for VRC01 from HEK cells. Furthermore, the neutralizing breadth and potency of plant‐produced VRC01 matched that of HEK‐cells‐produced VRC01 across a panel of HIV‐1 virus and pseudoviruses from four different clades. This was also a first demonstration of VRC01 production in stably transformed, transgenic lines, a manufacturing platform which is readily transferable to resource‐poor regions, and for which a regulatory pathway for cGMP compliant production has already been established in Europe (EMEA, 2008).

## Experimental procedures

### Cloning of VRC01 heavy and light chain genes

The heavy (gamma) and light (kappa) chain genes of the broadly neutralizing antibody VRC01 were plant‐codon‐optimized using GeneArt proprietary software (Geneart, Regensburg, Germany) and provided by MAPP Biopharmaceuticals (San Diego, CA).

For cloning into the binary expression vector pTRAk.2 (Sack *et al*., 2007; van Dolleweerd *et al*., in preparation), the genes were amplified using the common forward primer 5′GCGCCC**ATG**GCTAACAAGCACCTGTCT‐3′ and the reverse primer 5′GCGCTCTAGA**CTA**GCACTCTCCCCTATTAAAAG‐3′ (for light chain) or 5′GCGCTCTAGA**CTA**CTTACCAGGAGACAGAGAC‐3′ (for heavy chain). Restriction sites used for cloning the gene in to pTRAk.2 are underlined and the start/stop codons are in bold. The amplified PCR products were cloned into BsmBI site (for heavy chain) or BsaI site (for light chain) of a single pTRAk.2 vector using NcoI and XbaI.

### Stable transformation of *Nicotiana tabacum*

Stable transformation of *N. tabacum* leaf discs was performed as described by Horsch *et al*. (1985). Briefly, *Agrobacterium tumefaciens* strain GV3101 : pMP90(RK) transformed with pTRAk.2 harbouring VRC01 light and heavy chain genes was grown as above. After removal of medium by centrifugation, the pellet was resuspended in liquid MS (Murashige and Skoog, 1962) medium (Sigma, Gillingham, UK). Leaf discs (1 cm^2^) of 6‐week‐old *N. tabacum* cv Petit Havana SR‐1 grown under sterile conditions were immersed in the bacterial suspension for 20 min. The leaf discs were then incubated for 2 days on shoot‐inducing medium (SIM) consisting of MS medium, 0.1 μg/mL α‐naphthaleneacetic acid (NAA; Sigma) and 1 μg/mL benzylaminopurine (BAP; Sigma) at 23 °C. The leaf discs were then transferred to SIM with 500 μg/mL carbenicillin (Apollo Scientific, Stockport, UK) to eliminate the *Agrobacterium* and 100 μg/mL kanamycin to select for transformed cells. Regenerated shoots were rooted on MS medium with carbenicillin and kanamycin. Rooted plantlets were transferred to soil. Yields of rooted primary transformants (T_0_) were determined by ELISA (see below) of plant crude extracts obtained from two leaf discs. Three of the highest yielding T_0_ plants were allowed to self‐pollinate to obtain the T_1_ generation.

The highest yielding T_0_ plant was also repropagated by tissue culture to obtain purified VRC01 antibody. Briefly, leaves of the plant were sterilized for 15 min in 15% sodium hypochlorite. 1‐cm^2^ leaf discs were cultured in SIM with 500 μg/mL carbenicillin and 100 μg/mL kanamycin. Regenerated shoots were rooted on MS medium before being transferred to soil. Leaves of the regenerated plants were harvested for protein purification after 2.5–3 months just before flowering.

### Transient transformation of *Nicotiana benthamiana*

Vacuum infiltration of *N. benthamiana* leaves was performed as described by Kapila *et al*. (1997). *Agrobacterium tumefaciens* strain GV3101 pMP90RK transformed with pTRAk.2 harbouring VRC01 light and heavy chain genes was grown overnight in Luria‐Bertani (LB) broth, 0.02 mm acetosyringone (Santa Cruz Biotechnology, Dallas, TX), 0.01 mm MES (Sigma), 100 μg/mL rifampicin (Apollo Scientific) and 50 μg/mL kanamycin (Apollo Scientific) at 28 °C. After removal of the medium by centrifugation, the pellet was resuspended in infection solution containing 0.1 mm acetosyringone, 0.01 mm MES (Sigma) and 0.01 mm MgCl_2_ (VWR International, Lutterworth, UK). The final OD_600_ of the bacterial suspensions was adjusted to 0.3. Fully expanded leaves of 3.5‐week‐old *N. benthamiana* plants were immersed in the bacterial suspension in desiccator attached to a vacuum system consisting of a vacuum pump (Vacuubrand GMBH, Brackley, UK). Vacuum was applied for 1 min at 100 mbar. The plants were then further grown in a controlled environment room at 25 °C with 16/8 h light/dark cycle.

### Western blot detection of VRC01 expression in plant leaves

Two leaf discs collected from transformed plants were homogenized with three volumes of 100 mm Tris (pH 8.0; Sigma), 150 mm NaCl (VWR International), 1 mm EDTA (VWR International, Leuven, Belgium) and 1 μm PMSF (Roche, Burgess Hill, UK) using 3.175 mm chrome steel ball bearings and a Mixer Mill MM400 (Retsch, Castleford, UK). 15 μL of total soluble extract was resolved on NuPage 12% Bis‐Tris gel (Life Technologies, Paisley, UK) and transferred onto a nitrocellulose membrane. The membrane was blocked with blocking buffer [50 mm Tris (pH 7.5), 150 mm NaCl, 1% Tween‐20 (Sigma), 5% skimmed milk] and incubated with 1 : 10 000 peroxidase‐conjugated goat anti‐human kappa light chain antibody (Sigma, Gillingham, UK) to detect the light chain or 1 : 10 000 peroxidase‐conjugated polyclonal goat anti‐human IgG (Fc fragment) antiserum (Jackson Immunoresearch, West Groove, CA) to detect the heavy chain. Detection was performed using the ECL Prime system (Pierce, Rockford, IL) and visualized using G:Box F3 (Syngene, Cambridge, UK) or Amersham Hyperfilm ECL (GE Healthcare, Chalfont St Giles, UK).

### Immunoabsorbent assays (ELISA)

Five microgram per millilitre sheep anti‐human IgG gamma heavy chain Fc region (The Binding Site, Birmingham, UK) or 1 μg/mL gp120 IIIB (NIH ARRRP) was coated for 2 h at 37 °C. The plates were washed with three washes of wash buffer (water with 0.01% Tween‐20). The plates were blocked with PBS with 5% skimmed milk powder at 37 °C for 1 h. After three washes with wash buffer, the antibody samples (as crude plant extracts or pure protein) were incubated at 37 °C for 2 h (all dilutions were done in blocking solution). The plates were washed three times with wash buffer. A 1 : 1000 dilution of peroxidase‐conjugate goat anti‐human kappa light chain (Sigma) was added for 1 h. Bound antibodies were detected using TMB substrate solution (3,3′,5,5′‐tetramethylbenzidine (Sigma) in 25 mm citrate‐50 mm phosphate buffer, pH 5.0). The colour reaction was stopped by addition of 2 m sulphuric acid, and the absorbance was determined at 450 nm using a Sunrise plate reader (Tecan, Theale, UK). Titrations of known human IgG1 kappa (Sigma) were used as standards to determine antibody concentration.

### Protein purification

Vacuum infiltrated leaves were harvested at 6 days postinfection (dpi) for transient plants or just before flowering for stable plants. The leaves were extracted with three volumes of extraction buffer (100 mm Tris (pH 8.0), 150 mm NaCl, 1 mm EDTA and 1 μm PMSF). The clarified crude extracts were purified using a mixed protein G‐Sepharose/protein A‐agarose (Sigma) column. The column was washed with 10 column volumes (CV) of binding buffer (20 mm sodium phosphate, pH 7.0) and the protein eluted with elution buffer (0.1 m glycine‐HCl, pH 2.7). The eluted fraction was neutralized with 1 m Tris–HCl, pH 9.0 and then by buffer‐exchange diafiltration using Amicon Ultra‐15 (Molecular cut‐off 100 kDa; Milipore, Ireland) or by dialysis using Slide‐A‐Lyzer Cassettes (Molecular cut‐off 3.5 kDa; Thermo Scientific, USA) depending on the volume. Purified antibody concentration was determined by direct ELISA using titrations of known human IgG kappa (Sigma) as standards, reconfirmed with resolving titrations of purified antibodies against known concentrations of human IgG kappa on a NuPage 4%–12% Bis‐Tris Coomassie gel (Life Technologies) and either with A280 measurements using NanoDrop 2000 (Thermo Scientific, USA) or with BCA protein assay (Pierce, USA) using known concentration of bovine serum albumin (BSA) as standards.

### Glycopeptide analysis using LC‐ESI‐MS

Purified antibodies were reduced using 5 mm DTT in a 0.1 m ammonium bicarbonate buffer at 56 °C for 45 min followed by *S*‐carbamidomethylation using 25 mm iodoacetamide in a 0.1 m ammonium bicarbonate buffer at 25 °C for 30 min. After precipitation using 80% acetone at −20 °C for 45 min, the protein was digested with 0.2 μg trypsin in 0.1 m ammonium bicarbonate buffer overnight at 37 °C. After deactivation of trypsin at 96 °C for 6 min, the protein was digested with 0.2 μg endoproteinase GluC in 0.1 m ammonium bicarbonate buffer overnight at 37 °C. Glycopeptide analysis was performed using liquid chromatography–electrospray ionization–mass spectrometry (LC‐ESI‐MS) as described previously (Pabst *et al*., 2012) with a few modifications: the gradient was developed from 1% to 80% acetonitrile over 60 min at a flow rate of 6 μL/min, using 0.3 formic acid buffered to pH 3.0 with ammonia as the aqueous solvent. Peptide identification was performed with maXis 4G ETD (Bruker, Germany) in positive ion mode (ion cooler transfer time: 100 μs, ion cooler prepulse storage time: 10 μs; low mass: 300.00 m/*z*, spectra rate: 1 Hz).

### Surface plasmon resonance of antibody binding

The binding kinetics of plant‐ and HEK‐cells‐produced VRC01 to HIV‐1 gp120 IIIB were determined using a Protein A capture assay (Floss *et al*., 2009). The assay was performed on a BIAcore X‐100 instrument (GE healthcare, Chalfont St Giles, UK) at 25 °C with buffer HBS‐EP+ (10 mm HEPES, pH 7.4, 150 mm NaCl, 3 mm EDTA and 0.05% surfactant P‐20). Protein A (Sigma) was immobilized onto a CM5 chip to 3988 response units (RUs) with standard amine coupling. Purified VRC01 from plant or HEK cells was diluted in HBS‐EP+ buffer to yield a similar capture level of 100 RUs. gp120 IIIB was injected at a concentration of 20 μg/mL for 3 min at the flow rate of 30 μL/min and allowed to dissociate for another 7.5 min before regeneration with 10 mm HCl for 1 min at the flow rate of 10 μL/min. Referenced and blanked sensorgrams were fitted with BIAcore Evaluation software using a 1 : 1 Langmuir model of binding. Each assay was performed in triplicate. The relative activity (relα) of the plant‐derived VRC01 was calculated in relation to the HEK‐cells‐derived VRC01 using the equation described by Floss *et al*. (2009) with slight modifications: the gp120 binding response (*R*_t_) was taken at 190 s after the start of injection.

### HIV‐1 neutralization assays

HIV‐1 BaL neutralization assay was performed as described by Wei *et al*. (2003) and Montefiori (2004). Briefly, 200 TCID_50_ of HIV‐1 BaL was incubated with dilutions of purified plant‐ or HEK‐cells‐produced VRC01 for 1 h at 37 °C in a total volume of 100 μL. Plant‐ and HEK‐cells‐produced VRC01 were titrated twofold seven times in triplicate starting at 10 μg/mL. All dilutions were performed in growth medium [DMEM with 10% foetal calf serum (FCS), penicillin–streptomycin (100 units/mL and 100 μg/mL, respectively) and 2 mm L‐glutamine]. The mixture were then added to 100 μL TZM‐bl cells expressing CD4, CCR5 and firefly luciferase gene under the control of HIV long‐terminal repeat sequence grown in growth medium preseeded at a concentration of 5 × 10^5^ cells/mL. Cells plus virus positive control and a cells only background control were also added. After a 24‐h incubation in 37 °C, 5% CO_2_ followed by addition and incubation with lysis buffer, the cell lysate was removed from each well and mixed in a 1 : 1 ratio with Bright Glo reagent (Promega, Southampton, UK) in a 96‐well white solid plates for luminescence measurements. The percentage reduction in relative light units (RLU) was calculated relative to the RLU of the ‘virus plus cells’ positive control. The resulting curve was plotted and analysed in GraphPad Prism.

HIV‐1 Env‐pseudovirus neutralization assay measures reduction of luciferase expression following a single round of pseudovirus infection (Li *et al*., 2005). Stocks of Env‐pseudovirus were prepared by transfection of 293T/17 cells. Plant‐ and HEK‐cells‐produced VRC01 were titrated fivefold seven times in duplicate starting at 10 μg/mL. 200 TCID_50_ of pseudovirus was added with the antibodies and incubated for 1 h at 37 °C. All other conditions of the assay are as per those described with the HIV‐1 BaL but with a 48‐h incubation time instead of 24 h. Both plant‐ and HEK‐cells‐produced VRC01 were tested against 3 Clade B (SC422661.8, PVO.4, AC10.0.29), 3 Clade C (Du156.12, ZM109.F.PB4, ZM53M.PB12), 3 Clade A (3415.v1.c1, 3365.v2.c20, 0260.v5.c1) and 1 Clade G (X1193_c1) pseudoviruses.

### Statistical methods

All statistical analyses were performed using GraphPad Prism 6 software (GraphPad, San Diego) by one‐way analysis of variance (ANOVA) with the significance level set at *P *<* *0.05. This is followed by Tukey–Kramer *post hoc* analysis.
